# Mitigation of water stress in broccoli by soil application of humic acid

**DOI:** 10.1038/s41598-024-53012-4

**Published:** 2024-02-02

**Authors:** Ehab A. Ibrahim, Noura E. S. Ebrahim, Gehan Z. Mohamed

**Affiliations:** Vegetables Research Department, Horticulture Research Institute, Agricultural Research Center, 9 Cairo University St., Orman, Giza, Egypt

**Keywords:** Drought, Environmental impact

## Abstract

The main challenge to plant productivity is water scarcity, which is predicted to get worse with climate change, particularly in arid and semi-arid areas. Humic acid could improve plant tolerance to mitigate drought damage, which is an effective strategy to improve crop production and agriculture sustainability under limited water conditions in these regions, but its effective application rates should also be established. Thus, two field experiments were carried out at the Qaha Vegetable Research Farm in Qalubia Governorate, Egypt, during the two seasons of 2020–21 and 2021–22 on clay soil. The present study investigated the effect of three rates of humic acid application (0, 4.8, and 9.6 kg ha^-1^) on growth, yield, and quality of broccoli cv. Montop F1 hybrid under well-watered and drought conditions. Drought was induced by missing alternate irrigation. Soluble humic acid as potassium-humate was applied three times with irrigation water at the time of the first three irrigations of drought treatment. Water-stressed plants had a decrease in growth, yield, leaf chlorophyll, and nutrient content, while they showed an increase in the contents of leaf proline and curd dry matter and total soluble solids as well as water use efficiency, in both seasons. Soil application of humic acid was effective in mitigating the adverse effects of water deficit stress on the growth and yield of broccoli. Water-stressed plants had the highest WUE value (9.32 and 9.36 kg m^3−1^ in the first and second seasons, respectively) when the maximal humic acid rate was applied. Humic acid at a high level (9.6 kg ha^−1^) had the most promising results and represents an opportunity that must be applied to improve broccoli yield and its production sustainability in arid and semiarid regions.

## Introduction

Broccoli (*Brassica oleracea* var. *italic* Plenck) is considered one of the major vegetable crops belonging to the *Brassicaceae* family. The world production of broccoli and cauliflower in 2022 was about 25 million tons harvested from about 1.4 million hectares^1^. Broccoli has many benefits for human health because it is an important source of health-promoting compounds that possess anticancer, antioxidant, anti-microbial, and anti-inflammatory and is rich in the contents of minerals (such as Ca, Fe, Zn, and Mg), vitamins (such as A, C, niacin, and thiamine), phenolic compounds, glucosinolates, and fibers^2,3^.

The scenarios of global climate change have increased the intensity of abiotic stress. Water stress has been becoming an important issue because it is one of the greatest abiotic stressors affecting sustainable crop production^4^, which usually results in high growth, yield, and quality losses^5^. Egypt has been classified as a climate change hotspot. Thus, water scarcity is one of Egypt's most pressing issues^6,7^. More than 70% of Egypt's agricultural land is irrigated by inefficient surface irrigation systems, which result in high water losses, decreased land productivity, waterlogging, and salinity issues due to intensive irrigation and poor drainage^8^.

The most crucial factor affecting the yield and quality of broccoli is irrigation. Excessive irrigation led to water and nutrient losses through deep percolation below the root zone and low plant growth and yield. On the other hand, inadequate irrigation causes water stress and reduces growth and yield^5,9^, because the broccoli plant is shallow-rooted and sensitive to water stress. However, an economic income from broccoli production can be obtained by saving 30% of water, but if water stress exceeds more than 30%, the growth, yield, and quality of broccoli can be significantly lost^10,11^. Hence, there is a need to enhance broccoli cultivation in areas with water stress, such as semi-arid areas where Egypt is located.

Lately, different strategies have been considered as a potential approach to maintain the plant’s growth, yield, as well as quality under water stress. Humic acid has received increasing attention as a potential soil amendment for increasing crop growth and production. It has been demonstrated to promote plant growth and yield in normal conditions. Researchers have concentrated on humic acid because it can play beneficial roles in soils and plants due to its non-degrading nature and ability to resist microbial responses^12^. Humic acid can be applied to soil to improve it or stimulate plant growth. It enhances the physical, chemical, and biological properties of the soil, including the ion exchange capacity, nutrient availability, and water retention capacity^12–14^.

The soil application of humic acid is one method that may reduce irrigation and improve water use efficiency, as well as stimulate plant growth and productivity under water stress and no-stress conditions^9,12^. Humic acids also enhance plant hormones such as cytokinin and auxin, which are necessary for nutrient metabolism, photosynthesis, and stress tolerance in plants^15,16^. It's been reported that humic acid rates perform best under abiotic stress conditions such as water deficits^12,17^. Humic acid has antioxidant properties that inhibit the production of reactive oxygen species (ROS) and protect cells from oxidative harm^5,13^. As a result, it falls under the category of plant biostimulants.

Limited studies have been conducted on the effect of humic acid application on broccoli, such as those by El-Afifi et al.^9^ and Sakr et al.^5^, who indicated the role of humic acid in mitigating water stress in broccoli. Despite the rising interest in using humic acid in vegetable production systems, especially sustainable low-input systems; there is a lack of research on how different humic acid levels affect the growth, production, and quality of broccoli plants at various irrigation levels. Because application rates of humic acid depend on crop type and environmental and soil conditions^12^, it is challenging to forecast how different crops will respond to humic acid. Thus, further research is still necessary on the suitable level of humic acid doses under normal and deficit irrigation, especially in clay soil. The main goals of this study were to investigate how broccoli plants respond to soil applications of humic acid levels in terms of growth, yield, quality, and water use efficiency under either regular irrigation or water deficit irrigation.

## Results

### Vegetative growth

The deficit irrigation significantly reduced the vegetative growth characteristics in both seasons. When irrigation levels were kept constant, soil application of humic acid treatments had significant effects on plant fresh weight, plant height, number of leaves per plant, and leaf area per plant compared with the control treatment. The highest values of these parameters were recorded with normal irrigation at the highest level of humic acid. Meanwhile, the lowest values were recorded with untreated plants (control) under drought conditions (Table 1).

**Table 1:** Effect of the interaction between irrigation regimes and soil application of humic acid on broccoli vegetative growth during the 2020–21 and 2021–22 seasons. Values represent mean ± standard deviation. Means followed with similar letters within the same column are not different significantly at *P* < 0.05 level of probability based on the LSD test.

Treatments		Plant fresh wt (g)		Plant height (cm)		No. leaves plant^−1^		Leaf area (cm^2^ plant^−1^)	
Irrigation	Humic acid (kg ha^-1^)	2020–21	2021–22	2020–21	2021–22	2020–21	2021–22	2020–21	2021–22
Well-watered	0	1472 ± 53.0 c	1431 ± 39.0 c	72.1 ± 1.7 c	71.5 ± 1.9 c	20.2 ± 1.1 c	19.7 ± 0.6 c	7045 ± 162 c	6930 ± 124 c
	4.8	1586 ± 55.6 b	1555 ± 47.0 b	75.4 ± 1.9 b	74.5 ± 1.4 b	21.3 ± 0.9 b	20.9 ± 0.4 b	7483 ± 203 b	7245 ± 120 b
	9.6	1709 ± 57.0 a	1691 ± 47.5 a	79.3 ± 1.8 a	78.2 ± 1.0 a	22.5 ± 0.6 a	22.1 ± 0.3 a	7842 ± 203 a	7653 ± 148 a
Drought	0	1134 ± 41.0 f	1120 ± 30.5 f	57.3 ± 1.2 f	56.8 ± 1.9 f	15.6 ± 0.4 f	15.4 ± 1.1 f	4389 ± 103 f	4147 ± 72 f
	4.8	1269 ± 46.5 e	1212 ± 34.5 e	63.6 ± 1.4 e	62.4 ± 1.6 e	17.1 ± 0.7 e	16.7 ± 0.8 e	5425 ± 139 e	5125 ± 62 e
	9.6	1306 ± 45.5 d	1326 ± 43.2 d	68.7 ± 1.7 d	67.8 ± 1.8 d	18.7 ± 0.8 d	18.3 ± 0.7 d	6464 ± 161 d	6296 ± 102 d
LSD		6.8	12.9	0.43	0.63	0.18	0.60	79.3	51.0

### Leaf N, P, and K concentrations

**Figure 1:**
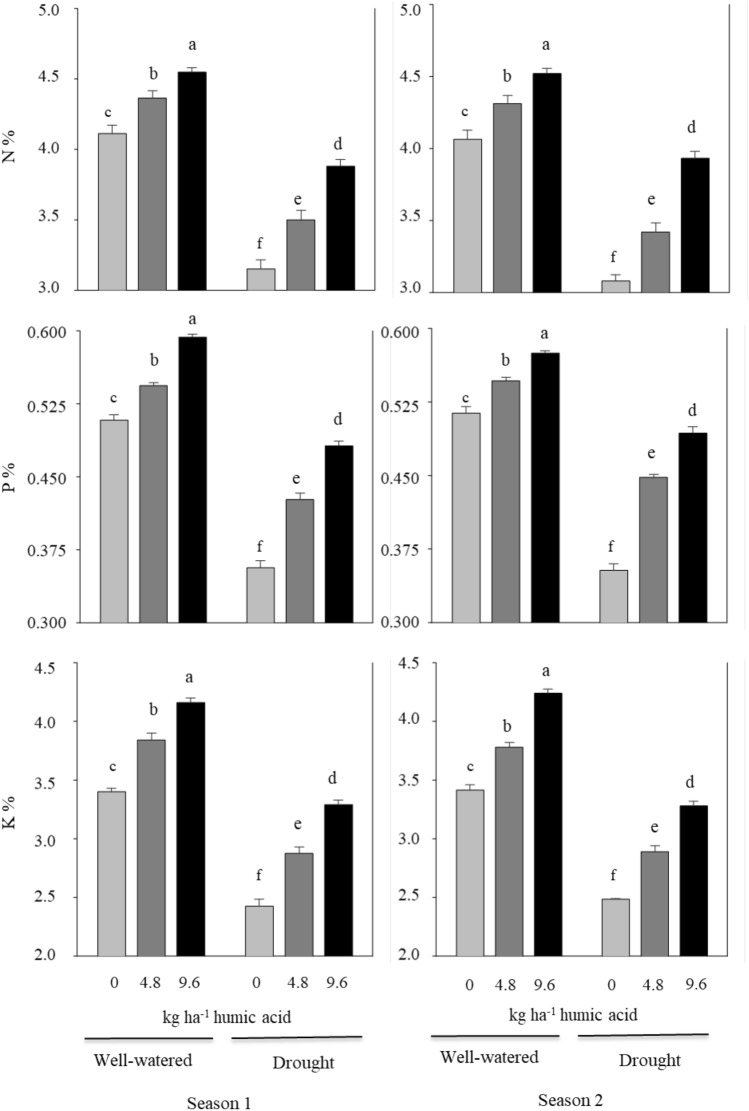
Effect of the interaction between irrigation regimes and soil application of humic acid on N, P, and K concentrations in broccoli leaves during the 2020–21 and 2021–22 seasons. Data are presented as the means ± SD (n = 3). Different letters mean significant differences among the treatments at *P* < 0.05 level.

The normal irrigation treatment had the highest values of N, P, and K concentrations as compared to the deficit irrigation treatment (Fig. 1). These parameters were also significantly increased by applying humic acid treatments as compared with the control treatment when irrigation levels were kept constant. The maximum values of the N, P, and K concentrations were observed in the application of humic acid at 9.6 kg ha^−1^ under normal irrigation in comparison with other interaction treatments. Meanwhile, the lowest values of N, P, and K concentrations were obtained from drought-stressed plants without any soil application of humic acid. Similar results were obtained in both seasons.

### Leaf chlorophyll and proline content

**Figure 2:**
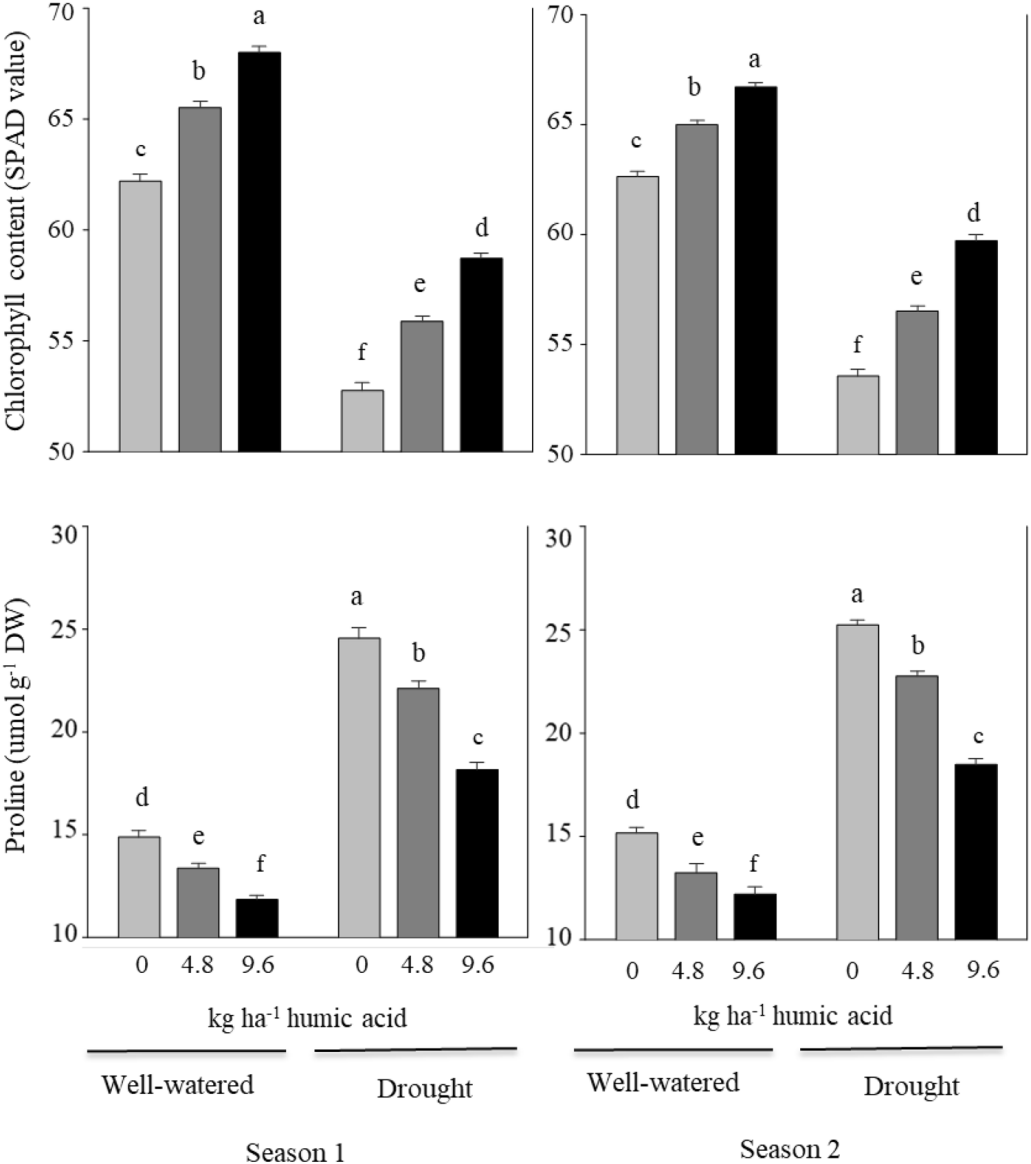
Effect of the interaction between irrigation regimes and soil application of humic acid on chlorophyll and proline content in broccoli leaves during the 2020–21 and 2021–22 seasons. Data are presented as the means ± SD (n = 3). Different letters mean significant differences among the treatments at *P* < 0.05 level.

Comparing the values of the interaction between the two factors under study revealed that, at any irrigation regime, increasing humic acid rates increased chlorophyll content and decreased proline content in both seasons (Fig. 2). The treatment combination of humic acid at 9.6 kg ha^−1^ and normal irrigation recorded the highest values of chlorophyll content and the lowest values of proline content. On the other hand, the combined treatment of humic acid at 0 kg ha^−1^ and drought recorded the lowest values of chlorophyll content and the highest values of proline content in both seasons.

### Curd quality characters

Limited irrigation caused significant increases in total soluble solids (TSS) content and dry matter percentages of broccoli curds, while main and secondary curd weights were reduced in both seasons (Table 2). On the other hand, the soil application of humic acid significantly enhanced the main and secondary curd weight and significantly decreased the TSS and dry matter percentage under both irrigation regimes in both seasons. The highest values of main and secondary curd weight were recorded by the humic acid application in non-stressed plants, but the highest values of TSS and dry matter percentage were recorded in stressed plants without humic acid application in both seasons. Meanwhile, the lowest values of main and secondary curd weight were obtained from untreated plants under limited irrigation conditions in both seasons. The lowest values of TSS and dry matter percentage were achieved in non-stressed plants with the application of a higher rate of humic acid in both seasons.

**Table 2:** Effect of the interaction between irrigation regimes and soil application of humic acid on curd quality characters of broccoli during the 2020–21 and 2021–22 seasons. Values represent mean ± standard deviation. Means followed with similar letters within the same column are not different significantly at *P* < 0.05 level of probability based on the LSD test.

Treatments		Main curd weight (g)		Secondary curd weight (g)		Dry matter in curd (%)		TSS in curd (%)	
Irrigation	Humic acid (kg ha^−1^)	2020–21	2021–22	2020–21	2021–22	2020–21	2021–22	2020–21	2021–22
Well-watered	0	484 ± 17.5 c	451 ± 24.5 bc	139 ± 5.5 b	136 ± 4.0 c	10.92 ± 0.3 d	10.71 ± 0.2 d	7.63 ± 0.1 d	7.73 ± 0.1 d
	4.8	506 ± 20.5 b	475 ± 20.1 ab	146 ± 5.0 a	142 ± 3.0 b	10.28 ± 0.3 e	10.14 ± 0.2 e	7.40 ± 0.1 e	7.47 ± 0.1 e
	9.6	525 ± 11.0 a	496 ± 11.0 a	150 ± 3.5 a	146 ± 2.0 a	9.62 ± 0.3 f	9.55 ± 0.2 f	7.30 ± 0.1 f	7.40 ± 0.1 f
Drought	0	338 ± 18.0 f	320 ± 20.5 e	92 ± 6.1 e	92 ± 6.5 f	12.91 ± 0.1 a	12.78 ± 0.1 a	8.17 ± 0.1 a	8.27 ± 0.1 a
	4.8	385 ± 14.0 e	374 ± 23.5 d	115 ± 3.0 d	117 ± 5.0 e	11.98 ± 0.1 b	11.86 ± 0.1 b	8.00 ± 0.1 b	8.10 ± 0.1 b
	9.6	438 ± 14.3 d	428 ± 21.0 c	127 ± 4.0 c	125 ± 5.0 d	11.41 ± 0.2 c	11.28 ± 0.2 c	7.77 ± 0.1 c	7.90 ± 0.1 c
LSD		8.5	36.6	5.6	2.6	0.09	0.06	0.07	0.07

### Yield and its components

The irrigation water deficit significantly reduced the main, secondary, and total yield of broccoli curds in comparison with well-watered plants in both seasons (Table 3). Soil application of humic acid treatments had a significant effect on broccoli yield and its components compared to control treatments under the same irrigation regime in both seasons. The highest significant values for main, secondary, and total yield were obtained with the application of humic acid at 9.6 kg ha^−1^ to well-watered plants. Meanwhile, the lowest values of yield and its components were obtained from untreated plants under drought conditions. Similar results were obtained in both seasons.

**Table 3:** Effect of the interaction between irrigation regimes and soil application of humic acid on yield and its components of broccoli during the 2020–21 and 2021–22 seasons. Values represent mean ± standard deviation. Means followed with similar letters within the same column are not different significantly at *P* < 0.05 level of probability based on the LSD test.

Treatments		Total main curd yield (ton ha^−1^)		Total secondary curd yield (ton ha^−1^)		Total yield (ton ha^−1^)	
Irrigation	Humic acid (kg ha^−1^)	2020–21	2021–22	2020–21	2021/22	2020–21	2021–22
Well-watered	0	12.484 ± 0.31 c	11.971 ± 0.30 c	11.664 ± 0.21 c	11.350 ± 0.22 c	24.148 ± 0.51 c	23.321 ± 0.52 c
	4.8	13.255 ± 0.21 b	12.858 ± 0.30 b	12.101 ± 0.15 b	11.825 ± 0.27 b	25.356 ± 0.36 b	24.683 ± 0.57 b
	9.6	13.955 ± 0.14 a	13.618 ± .35 a	12.753 ± 0.11 a	12.538 ± 0.30 a	26.708 ± 0.25 a	26.156 ± 0.64 a
Drought	0	9.282 ± 0.38 f	9.086 ± 0.11 f	8.585 ± 0.30 f	8.200 ± 0.11 f	17.867 ± 0.68 f	17.286 ± 0.26 f
	4.8	10.332 ± 0.38 e	10.000 ± 0.21 e	9.426 ± 0.24 e	9.092 ± 0.17 e	19.758 ± 0.62 e	19.091 ± 0.38 e
	9.6	11.611 ± 0.26 d	11.293 ± 0.29 d	10.430 ± 0.30 d	10.282 ± 0.22 d	22.041 ± 0.55 d	21.575 ± 0.51 d
LSD		0.20	0.16	0.19	0.14	0.29	0.30

### Seasonal applied water

Normal irrigation treatment resulted in the highest values of seasonal applied water with significant differences as compared to drought in both seasons (Table 4). As soil application of humic acid increased, seasonal applied water was significantly reduced under both limited and normal irrigation conditions in both seasons. The lowest values of seasonal applied water were obtained in plants grown under limited irrigation with the application of a higher rate of humic acid, and the values were 2364 and 2306 m^3^ ha^−1^ in the first and second seasons, respectively.

**Table 4:** Effect of the interaction between irrigation regimes and soil application of humic acid on seasonal applied water and water use efficiency of broccoli during the 2020–21 and 2021–22 seasons. Values represent mean ± standard deviation. Means followed with similar letters within the same column are not different significantly at *P* < 0.05 level of probability based on the LSD test.

Treatments		Total applied irrigation water (m^3^ ha^−1^)		Water use efficiency (kg m^3−1^)	
Irrigation	Humic acid (kg ha^−1^)	2020–21	2021–22	2020–21	2021–22
Well-watered	0	4228.0 ± 87.50 a	4186 ± 105.50 a	5.71 ± 0.01 f	5.57 ± 0.02 f
	4.8	4043.2 ± 107.00 b	3994 ± 100.50 b	6.27 ± 0.08 e	6.18 ± 0.01 e
	9.6	3837.6 ± 116.52 c	3716 ± 94.50 c	6.96 ± 0.15 c	7.04 ± 0.01 c
Drought	0	2746.4 ± 115.51 d	2718 ± 78.00 d	6.51 ± 0..03 d	6.36 ± 0.11 d
	4.8	2517.6 ± 98.50 e	2511 ± 43.30 e	7.85 ± 0.06 b	7.60 ± 0.08 b
	9.6	2364.0 ± 70.00 f	2306 ± 24.91 f	9.32 ± 0.06 a	9.36 ± 0.26 a
LSD		14.91	71.82	0.12	0.27

### Water use efficiency

The obtained results in Table 4 show that there are significant differences among the interaction treatments for water-use efficiency (WUE). The WUE values were significantly increased under deficit irrigation conditions in both seasons. Soil application of humic acid significantly increased WUE under limited and normal irrigation in both seasons. The highest WUE value was observed in plants grown under water stress when the maximum humic acid rate (9.6 kg ha^−1^) was applied, and the values were 9.32 and 9.36 kg m^1,3^ in the first and second seasons, respectively.

## Discussion

The results of this study showed that broccoli plants subjected to water stress had significantly lower vegetative growth and yield attributes than well-watered ones. These findings are supported by data provided by Erken et al.^18^, Durak and Yıldırım^10^, Hossain and Mohona^11^, El-Afifi et al.^9^, and Sakr et al.^5^. These increases under well-watered conditions could be the result of greater physiological processes, better nutrient uptake, and faster rates of photosynthesis, which could be reflected in more leaves and a larger leaf area as well as higher growth and yields^5,19,20^. On the other hand, water stress impairs crop growth and productivity by hampering physiological processes due to damage to cell membranes and photosystems. It has been demonstrated that during drought stress, plants lose chlorophyll and produce less photosynthetic products^21,22^. Water stress triggers oxidative stress on plants due to a decline in the activities of antioxidant enzymes, and generates ROS, which degrades cell membrane activities and restricts plant growth and production^22–24^. Plants often establish a defense mechanism against ROS that includes antioxidant enzymes and non-enzymatic compounds like proline^22,25,26^.

The results also showed that increasing humic acid application significantly promoted all studied growth and yield traits under both conditions and in both seasons. In particular, in situations of water stress, the prospective effect of applied humic acid was evident in improved broccoli growth and yield when applying 9.6 kg humic acid per hectare. Similar studies found that the application of humic acid had a significant increase in plant height, fresh weight, and total yield of secondary and main head heads of the broccoli plant, as well as N, P, and K content, total chlorophyll, dry matter, and TSS content compared to untreated plants^5,9,27–29^.

It has been reported that the humic acid application promoted broccoli growth and yield through nutrient uptake, photosynthesis, cell respiration, protein synthesis, and enzyme activities^30^. The positive effects of humic acid have been related to improving soil properties such as aeration, aggregation, water holding capacity, ion availability, and transport that lead to more effective nutrient and water uptake, and more accumulation of photosynthates even under water stress^12,14,31^. Furthermore, humic acid can stimulate protective antioxidant responses in plants to prevent the production of ROS and protect cells from oxidative damage^5,13^. Also, humic acid promotes cell division and lengthening by enhancing growth regulators, which positively impacts the stimulation of plant growth^12,16^.

The findings of the current study revealed that water deficit stress reduced the N, P, and K contents of broccoli leaves compared to non-stressed plants. The low levels of N, P, and K detected under drought may be due to decreased soil moisture levels and nutrient distribution caused by water deficit stress, which inhibited nutrient absorption and transfer^27^. However, humic acid applications prevent these negative effects with higher values of N, P, and K levels in water-stressed plants. These results are supported by data reported by Bhatt and Singh^32^, who found that the effect of humic acid depends on the level of doses; a higher rate gave maximum values in total nitrogen, available phosphorus, and potassium in the soil. In this respect, Bhatt and Singh^32^ reported that because humic acid has a large surface area as a part of the humus, it has a greater cation exchange capacity. Thus, it exchanges the nutrients from the soil, stores them in its molecules, and then gradually releases them as the plants require. There are numerous binding sites in humic acid for macronutrients like P, K, and Ca. Moreover, Ampong et al.^12^ reported that humic acid can stabilize ammonium and increase the soil's availability of nitrogen; moreover, plants can access nitrogen, which is also present in humic acid molecules. However, Al-jaf et al.^28^ found that the application of various humic acid dosages had no discernible impact on the NPK levels. The results of this study also showed that water deficit conditions reduced the chlorophyll content in leaves compared to non-stressed plants, resulting in reduced photosynthesis efficiency and plant growth. This finding is confirmed by data from other studies^5,25^. Drought negatively affects photosynthesis because it reduces leaf area and damages the photosynthetic machinery, including total chlorophyll content^33^. Moreover, the current study's results showed that the humic acid application significantly increased chlorophyll levels compared to untreated plants. Sakr et al.^5^ and Khorasani et al.^34^ reported similar results. The elevated chlorophyll levels in treated plants are most likely the result of improved plant absorption of nutrients, especially nitrogen (Fig. 1), which is essential for chlorophyll synthesis. Under conditions of water stress, proline is one of the osmoprotectants and ROS scavengers that build up in cells to protect against oxidative damage^35,36^, as confirmed by the current study and other studies^10,18,25,26^. Also, according to the current study, humic acid application caused a decrease in leaf proline levels in plants that were under well-watered and water-stress conditions (Fig. 2). Alsamadany^37^ also found significant decreases in proline content under water stress due to humic acid application. The results of this investigation showed that the dry matter and TSS contents of broccoli curd increased under conditions of water deficiency stress. Changes in metabolic traits are related to changes in plant tolerance to drought stress. When there is a water deficit, plant tissue will accumulate soluble carbohydrates as a protective measure. The buildup of dry matter and TSS may boost the plant's ability to tolerate drought stress^5,38^. It has been reported that humic acid application enhanced dry matter and TSS content^5,27–29^. Shah et al.^24^ stated that this is explained by humic acid's ability to reduce abiotic stress impacts. This indicated that humic acid could improve broccoli's tolerance to drought and reduce the negative effects of water stress by modifying physiological and biochemical processes.

The results of the present study showed that normal irrigation uses more seasonal irrigation water than deficit irrigation during the two growing seasons of broccoli. The increase in direct evaporation may be the cause of these increases. These results are in strong agreement with those of Durak and Yıldırım^10^ and El-Afifi et al.^9^. Moreover, the seasonal water applied decreased significantly after the soil application of humic acid. The same findings were reported by El-Afifi et al.^9^. Based on curd yield and irrigation water amount, deficit irrigation was determined to be the best irrigation regime in terms of WUE. In this regard, Durak and Yıldırım^10^ and El-Afifi et al.^9^ found that broccoli plants used irrigation water more effectively when less of it was applied. The current study's findings also demonstrated that WUE was increased due to rising humic acid rates, which enhanced water stored in the effective root zone. This result is in harmony with El-Afifi et al.^9^. In this respect, Bhatt and Singh^32^ reported that many compounds, such as hydrophilic, hydrophobic, macromolecules, and functional groups, can be found in humic acid. Humic acid's hydrophilic properties attract hydrogen ions, which increase the soil's water holding capacity. These data suggested that the addition of humic acid mitigated the negative effects of drought.

## Conclusions

One of the most crucial elements in broccoli production is water stress, which typically leads to significant yield and quality losses. Climate change has made water an increasingly valuable strategic resource. Under these conditions, the application of sustainable strategies to increase water use efficiency and reduce the effects of water stress is of high priority. The most promising of these strategies is the use of environmentally benign substances like humic acid. The results of this study demonstrated that water deficit conditions decreased the growth, yield, and quality of broccoli, while it enhanced proline and water use efficiency. Moreover, humic acid application to the soil, especially when done three times at a rate of 9.6 kg ha-1, had a significant impact on broccoli plants grown under regular and deficit irrigation. It had a notable effect on vegetative growth characters, nutrient content, yield attributes, and water use efficiency. Thus, soil application of humic acid could be a promising strategy to establish sustainable systems for broccoli production in clay soil, which will not only increase growth, yield, and WUE under regular irrigation but also significantly minimize the negative impact of drought conditions on broccoli plants in the arid and semiarid regions. Further studies are needed with various crops, soil types, and abiotic stresses to optimize the combined effect of mineral or organic fertilizers and different humic acid application rates on crop performance and soil quality parameters.

## Materials and methods

### Site description

**Figure 3:**
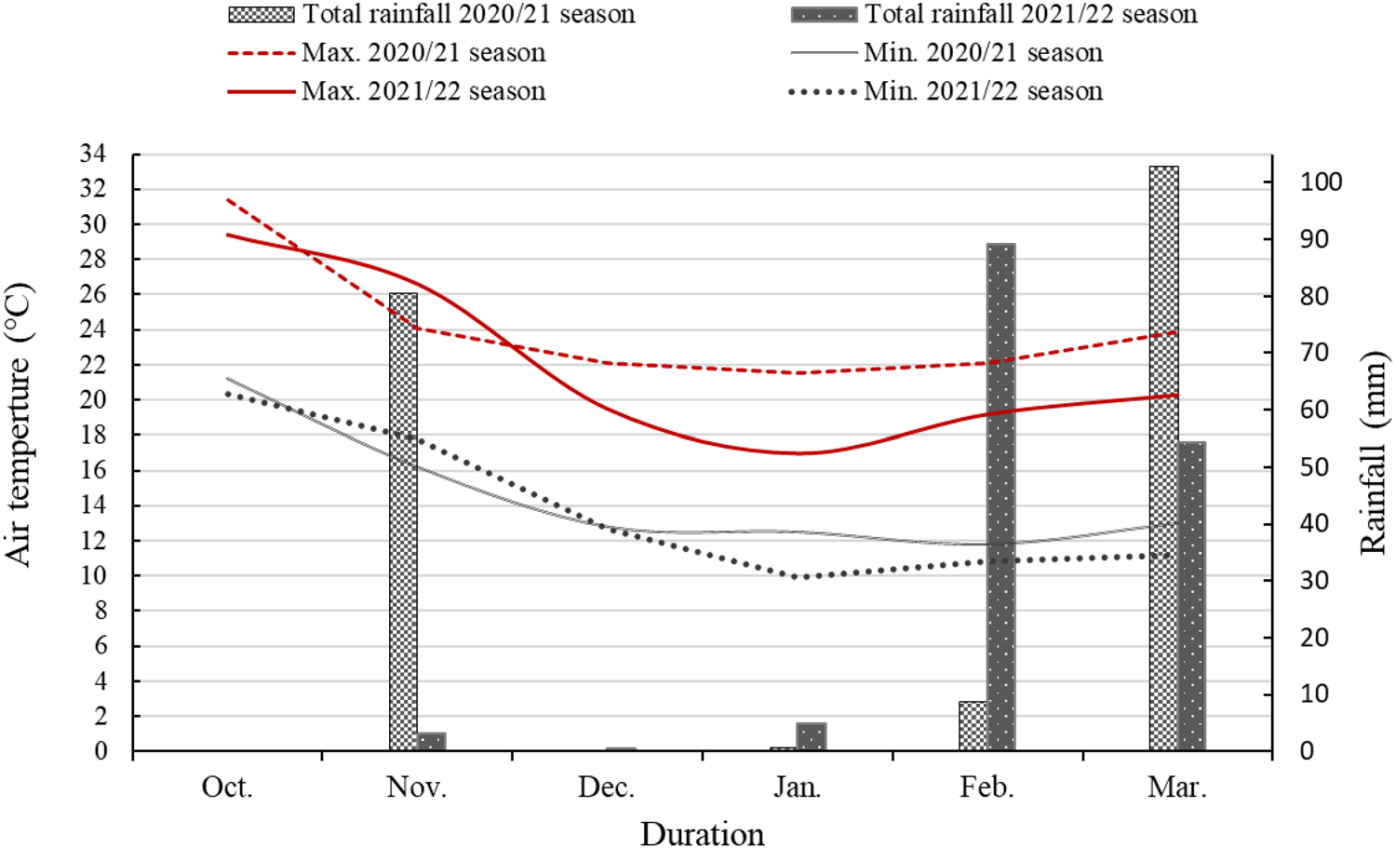
Monthly temperature and rainfall during the 2020–21 and 2021–22 seasons.

Two field experiments were conducted at the Qaha Vegetable Research Farm in Qalubia Governorate, Egypt (30°17′ 25" N, 31°15′ 50" E), during the winter seasons of 2020–21 and 2021–22. The averages of the annual high temperature, low high temperature, relative humidity, and average precipitation are 30.36 ºC, 16.81 ºC, 54.83%, and 2.1mm, respectively. The monthly averages of temperature and precipitation during the experimental seasons are shown in Fig. 3. These data were recorded by an automatic weather station of the Central Laboratory for Agriculture Climate, Egypt, at the experimental site. Soil samples were collected at random before the beginning of the experiments from a depth of 0–30 cm. The methods outlined by Page et al.^39^ and Klute^40^ were used to test the physical and chemical properties of experimental soil. The experimental soil was clay-textured and alkaline, with low organic matter and salt content. Table 5 provides details regarding soil properties.

**Table 5:** Physical, chemical, and hydrophysical analyses of the experimental soil before planting during the two winter seasons of 2020–21 and 2021–22.

Properties	Values		Properties	Values	
	2020–21	2021–22		2020–21	2021–22
Sand (%)	22.44	21.26	Available N (ppm)	67.65	70.98
Silt (%)	29.10	29.41	Available P (ppm)	5.27	5.79
Clay (%)	48.46	49.33	Exchangeable K (ppm)	59.53	58.64
Texture class	Clay	Clay	Field capacity (%)	46.09	45.82
CaCO3	2.62	2.49	Wilting point (%)	24.41	24.27
OM (%)	1.37	1.46	Available water (%)	21.68	21.55
pH (1:2.5 soil extract)	7.8	7.7	Bulk density (g cm^-3^)	1.22	1.21
EC (dSm^-1^) (1:10)	2.2	2.2			

### Experimental design and treatments

The experimental design was done using a split plot in randomized complete blocks with three replicates. Well-watered and drought treatments were arranged in the main plots, while, three different concentrations of humic acid (0, 4.8, and 9.6 kg ha^−1^) were randomly distributed in the subplots. Each experimental subplot contained five rows. Each row was 70 cm wide and 5 m long, with 50 cm between each plant. Transplants were placed on one side of the ridges. The treatments were separated by two guard ridges.

Surface furrow irrigation treatments were carried out after the initial irrigation, which was applied 10 days after the transplant. The well-watered treatment was irrigated every 10–12 days, according to the recommendation of Egyptian Ministry of Agriculture. In the drought treatment, missing alternate irrigation was applied, i.e., half of the irrigations were used compared to the well-watered treatment. For well-watered and drought treatments, the total number of irrigation events was 8 and 5, respectively. Good-quality irrigation water was used; it was characterized by pH EC (no more than 300 ppm). For humic acid treatments, soluble humic acid as potassium-humate (70% humic acid, 10% K_2_O, imported from Zhangjiagang Kangyuan New Material Co., Ltd., China by Art Chem Co., Kafr El-Zayat, Egypt) was applied three times with irrigation water at the time of the first three irrigations of water deficit treatment.

### Crop management

Seeds of broccoli cv. Montop F1 hybrid were sown in the nursery on September 1 in both seasons. The seedlings were transplanted when they were 45 days old. All treatments were fertilized with 144, 96, and 120 kg ha^−1^ of N, P_2_O_5_, and K_2_O in the form of ammonium sulfate, calcium superphosphate, and potassium sulfate, respectively. These fertilizers were applied in two equal doses; one was added after 21 days and the other after 40 days from transplanting. Other normal agricultural practices were followed until harvest.

### Data and measurements

The broccoli plants were harvested 75 days after transplanting. Five plants were randomly selected from each subplot, and the vegetative growth parameters of plant fresh weight, plant height, number of leaves per plant, and leaf area per plant with a portable leaf area meter (Li-3100, USA) were recorded. Samples of leaves were taken from recently expanded leaves of each plant to determine the N, P, and K percentages; they were ground after being oven-dried for 72 h at 60 °C. Nitrogen content was determined with the micro-Kjeldahl method^41^. Phosphorus was colorimetrically measured, while a flame photometer was used to determine potassium^42^. The middle portion of a young, fully grown leaf on five leaves per plot was chosen to measure the chlorophyll index using a handheld chlorophyll meter device (SPAD-502, Minolta, Sakai, Osaka, Japan). Five randomly selected recently expanded leaves from each plot were taken, wrapped in aluminum foil, and quickly transported to the laboratory to determine the proline content according to the method of Bates et al.^43^. Also, five samples of main curds were taken at random from each treatment to determine total soluble solids (TSS) and dry matter percentage. The hand refractometer method was used to determine TSS in broccoli curds. From each harvest and each treatment, the well-shaped and green curds were collected and weighed. The total main curd yield, total secondary curd yield, and total yield per hectare were recorded.

### Water relations

The difference in soil moisture content between before and after irrigation was used to calculate water consumption use, according to Israelson and Hansen^44^, as follows:

1 $${\text{Cu }} = {\text{ D }} \times {\text{ Bd }} \times { 1}0000 \, \times \, \left( {\theta_{{2}} - \, \theta_{{1}} } \right)/{1}00$$

where Cu is the water consumptive use m^3^ ha^−1^, D is the depth of the soil (30 cm), Bd is the soil bulk density (g cm^−3^), θ_1_ represents the soil moisture before irrigation (% by weight), and θ_2_ represents the soil moisture after 48 h from irrigation (% by weight).

The seasonal applied water is the sum of the figures obtained for each irrigation application. The water use efficiency was estimated for each treatment by dividing the yield (ton ha^−1^) by the total irrigation water given (m^3^ ha^−1^)^45^.

### Data analysis

Data were analyzed using the analysis of variance technique, and differences between individual pairs of treatment means were tested using the Least Significant Difference (LSD) at *p* < 0.05 by the Costat 6.29 computer program (CoHort Software), according to Snedecor and Cochran^46^. Also, the Number Cruncher Statistical System (NCSS) statistical program and Microsoft Excel® (2013) were used for the data analyses.

### Research involving plants statement

This study was developed with commercial seeds, therefore nonexotic or at risk of extinction, under controlled conditions, meeting all institutional, national and international guidelines and legislation for cultivated plants.

### Ethical approvals

This study was approved by The Ministry of Agriculture and Land Reclamation for permission to collect plant specimens.

## Data Availability

All data generated and/ or analyzed during this study are available from the corresponding author on reasonable request.
